# Accessory pathway unmasked by adenosine in a patient with unexplained cardiac arrest: a case report

**DOI:** 10.1093/ehjcr/ytaf451

**Published:** 2025-09-30

**Authors:** Simone Taddeucci, Luca Panchetti, Martina Nesti, Silvia Garibaldi

**Affiliations:** Department of Cardiology, Livorno Hospital, Viale Vittorio Alfieri, 36, Livorno 57124, Italy; Division of Cardiology, Tuscany Foundation ‘Gabriele Monasterio’, Via Moruzzi, 1, Pisa 56100, Italy; Division of Cardiology, Tuscany Foundation ‘Gabriele Monasterio’, Via Moruzzi, 1, Pisa 56100, Italy; Division of Cardiology, Tuscany Foundation ‘Gabriele Monasterio’, Via Moruzzi, 1, Pisa 56100, Italy; Division of Cardiology, Tuscany Foundation ‘Gabriele Monasterio’, Via Moruzzi, 1, Pisa 56100, Italy

**Keywords:** Unexplained sudden cardiac arrest, ST elevation, Adenosine test, Case report

## Abstract

**Background:**

Survivors of sudden cardiac arrest require detailed diagnostic evaluation to identify reversible causes and guide therapy. Underlying mechanisms may include ischaemia or electrophysiological abnormalities. The conventional diagnostic workflow may fail to uncover occult arrhythmic substrates, highlighting the need for targeted testing strategies in selected cases.

**Case summary:**

A 35-year-old man, a former endurance athlete, experienced a witnessed sudden cardiac arrest during sleep. Ventricular fibrillation was terminated with direct-current shock, achieving return of spontaneous circulation. The initial 12-lead ECG showed ST-segment elevation in V1–V3 with a coved Brugada-like pattern, but reciprocal ST-segment depression. Myocardial ischaemia was ruled out with coronary angiography, and Brugada syndrome was excluded by a negative ajmaline test. No further abnormalities were found. Persistent clinical suspicion prompted an adenosine test, which revealed a left lateral accessory pathway. Electrophysiological testing demonstrated high-risk properties of the accessory pathway, with haemodynamic collapse during pre-excited atrial fibrillation. Catheter ablation successfully eliminated the accessory pathway without changes in QRS complex morphology, and the patient remained asymptomatic at follow-up, with no arrhythmic recurrence.

**Discussion:**

Unexplained cardiac arrest in patients with structurally normal hearts presents significant diagnostic challenges. In this case, adenosine testing unmasked an accessory pathway as the reversible cause of cardiac arrest. Though not routinely included in cardiac arrest evaluations, adenosine testing was crucial in this patient’s diagnosis and management, preventing unnecessary implantable cardioverter–defibrillator placement. Comprehensive diagnostic strategies, including targeted use of adenosine testing, can reveal occult arrhythmic substrates, improving outcomes and avoiding overtreatment in selected cases.

Learning points

- The diagnostic workflow for apparently idiopathic VF is complex. Among other potential causes, signs of pre-excitation should be investigated through serial ECGs. Persistent clinical suspicion should prompt an adenosine test, which can be highly informative and guide the clinician to perform an electrophysiological study.
- Post ROSC ST elevation can be a non-specific sign and can complicate diagnosis.
- Adenosine administration test is a non-routine and non-invasive procedure that can reveal latent or subtle preexcitations.
- The identification of a reversible cause of sudden cardiac arrest in a patient with initial apparently idiopathic VF can avoid ICD implantation in young patients.

## Introduction

Survivors of sudden cardiac arrest (SCA) represent a high-risk population in whom identifying reversible causes through a structured diagnostic workflow is essential. The differential diagnosis is established through an approach that includes 12-lead electrocardiogram (ECG), multimodal imaging techniques, and invasive assessments such as coronary angiography. Some causes of SCA are reversible, while others require an implantable cardioverter-defibrillator (ICD) for secondary prevention. Continuous monitoring and personalized therapies are essential to improve the prognosis and reduce mortality.

We report the clinical case of a SCA in a young, apparently healthy patient with a high-risk left lateral accessory pathway (AP).

## Summary figure

**Figure:**
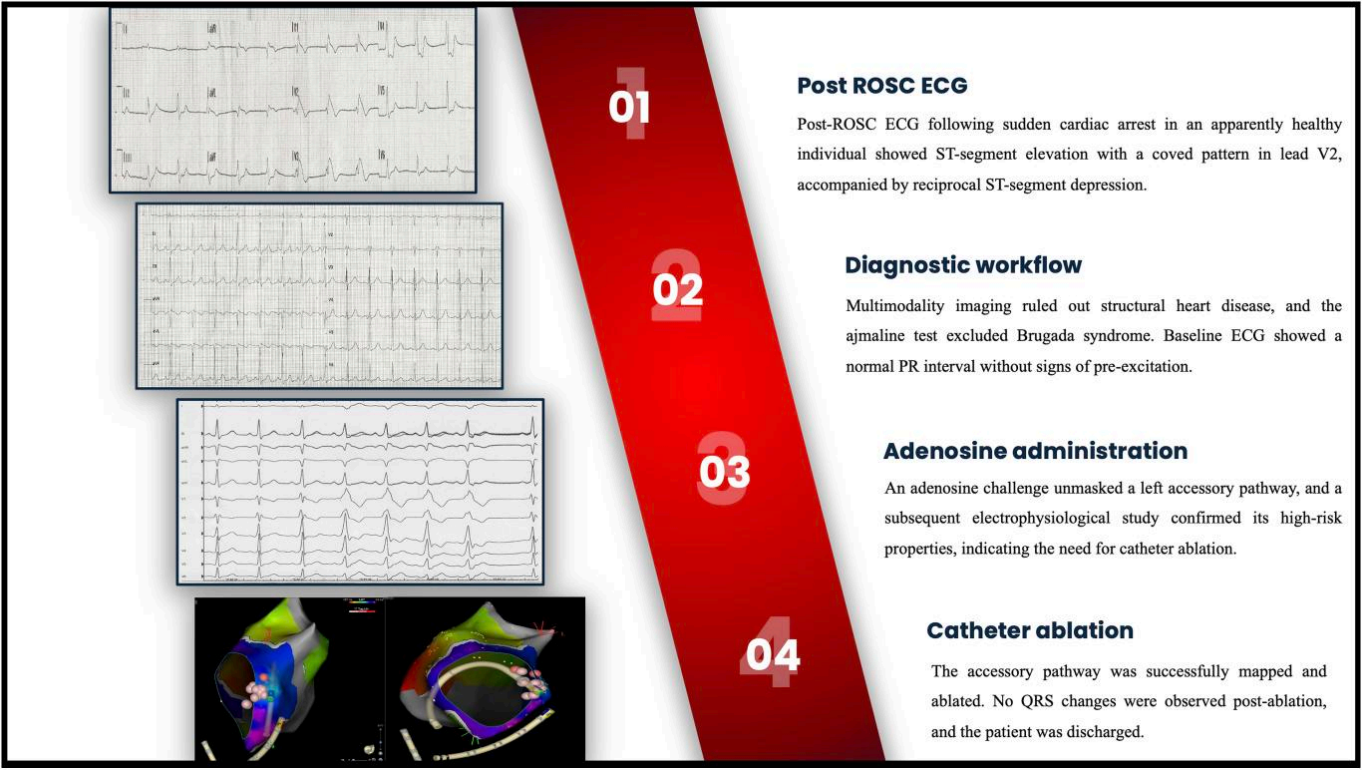


## Case presentation

**Figure 1:**
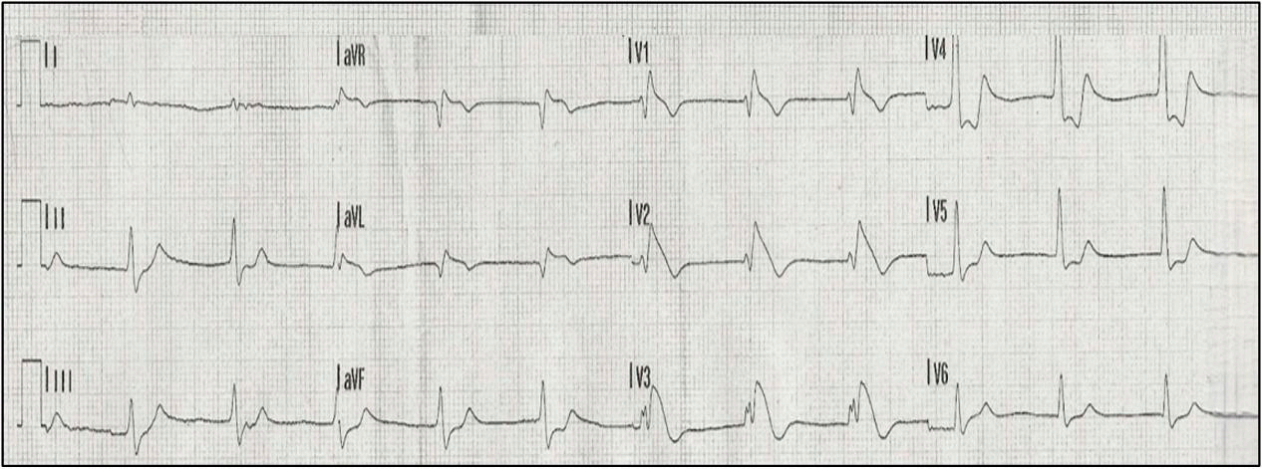
12-Lead ECG post ROSC.

A 35-year-old man with a history of ventricular extrasystoles, no family history of sudden cardiac arrest, and a background as a former endurance athlete experienced a cardiac arrest during sleep at 6:00 a.m. His wife witnessed the event, immediately began cardiopulmonary resuscitation manoeuvres, and called emergency services. At the first on-site medical evaluation, ventricular fibrillation (VF) was documented and resolved after the fourth direct-current shock. The patient was intubated and transferred to the nearest hospital. A 12-lead ECG performed 7 min after return of spontaneous circulation (ROSC) showed ST-segment elevation in the precordial leads V1–V3 with a ‘coved’ pattern, very similar to the Brugada pattern, but accompanied by reciprocal ST-segment depression in the inferior leads (*Figure 1*). P waves were not clearly identifiable on this initial ECG.

Upon hospital arrival, a coronary angiography excluded coronary artery disease or anatomical abnormalities. The patient was extubated and attained complete neurological recovery. Laboratory tests did not reveal any significant abnormality. After the coronary angiography, a second 12-lead ECG showed normalization of ST abnormalities, with only an incomplete right bundle branch block. The telemetry showed no arrhythmias nor dynamic ST-T changes. A bedside echocardiogram showed normal biventricular function and normal valvular apparatus. Cardiac magnetic resonance imaging confirmed these findings, with no evidence of structural disease or late gadolinium enhancement.

**Figure 2:**
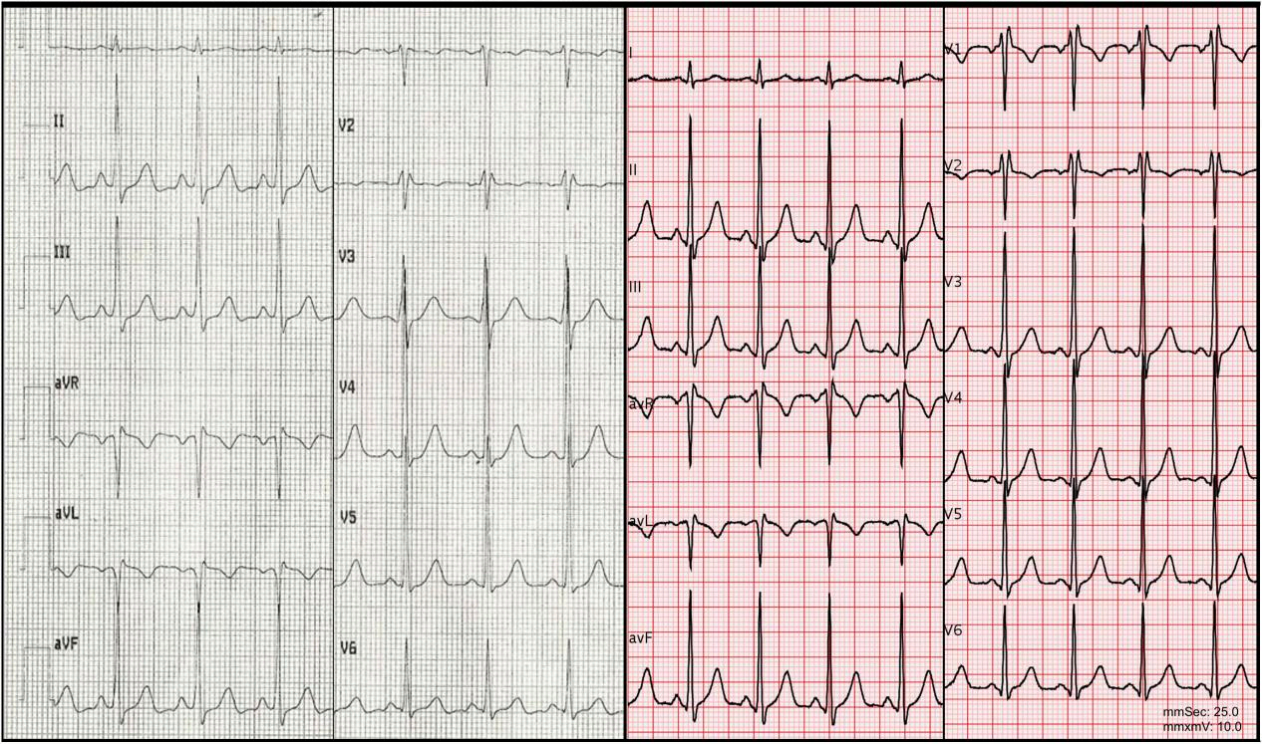
Two different ECGs were recorded after admission. The upstroke in V3 was slurred in both tracings; this observation could raise the hypothesis of a delta wave. However, the PR interval was normal, and physiological septal activation Q waves were present in the lateral leads on both ECGs. These findings were not sufficient to support a diagnosis of pre-excitation.

**Figure 3:**
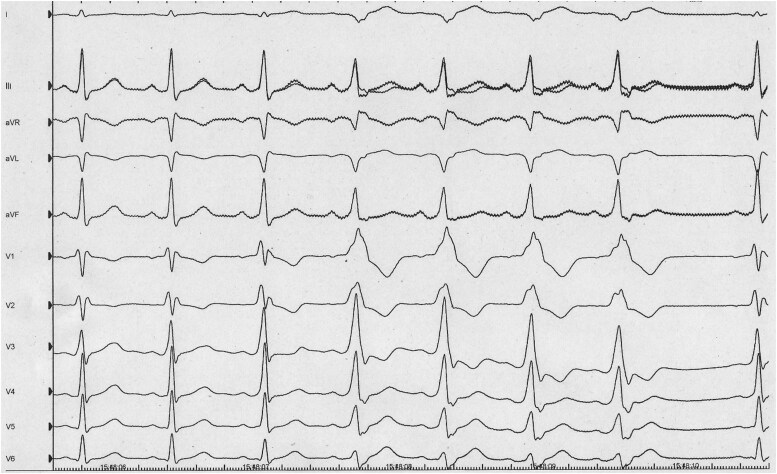
Twelve-lead ECG (25 mm/s) following administration of 18-mg intravenous adenosine.

Given the Brugada-like pattern on the 12-lead ECG obtained after ROSC, and the high-risk clinical scenario, an ajmaline test was performed, which provided negative results. Baseline ECGs (two tracings at *Figure 2*) showed a narrow QRS complex and a normal PR interval. Normal Q waves derived from physiological septal activation were present in the lateral leads. A slurred upstroke of the QRS in inferior leads and V3 was noted (*Figure 2*), yet with no evident delta wave. To exclude Wolff-Parkinson-White syndrome, an adenosine test was performed (i.v. bolus of adenosine 18 mg), and the temporary blockage of conduction through the atrioventricular node revealed the conduction over a left lateral AP (*Figure 3*). The fully pre-excited QRS showed right bundle block morphology, an inferior-rightward axis and positive concordance in the precordial leads, lacking the V5-V6 negative Q waves.

**Figure 4:**
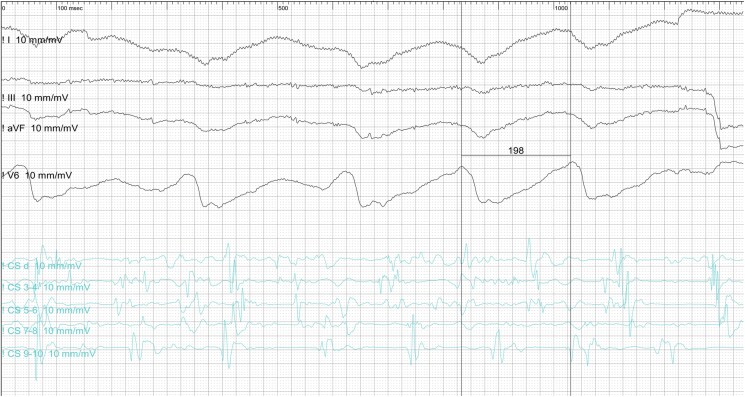
195 ms SPERRI in AF. CS: Coronary sinus.

Following this finding, an invasive electrophysiological study was conducted, confirming the presence of a left lateral AP with both antegrade and retrograde conduction. During programmed atrial stimulation under baseline conditions, the AP showed an antegrade refractory period of 200 ms. An orthodromic atrioventricular re-entrant tachycardia was induced and confirmed through entrainment manoeuvres. The iatrogenic induction of atrial fibrillation (AF) caused a wide-complex tachycardia with the shortest pre-excited R-R interval (SPERRI) of 196 ms during AF (*Figure 4*). This caused haemodynamic collapse, requiring immediate cardioversion, which restored sinus rhythm and consciousness.

**Figure 5:**
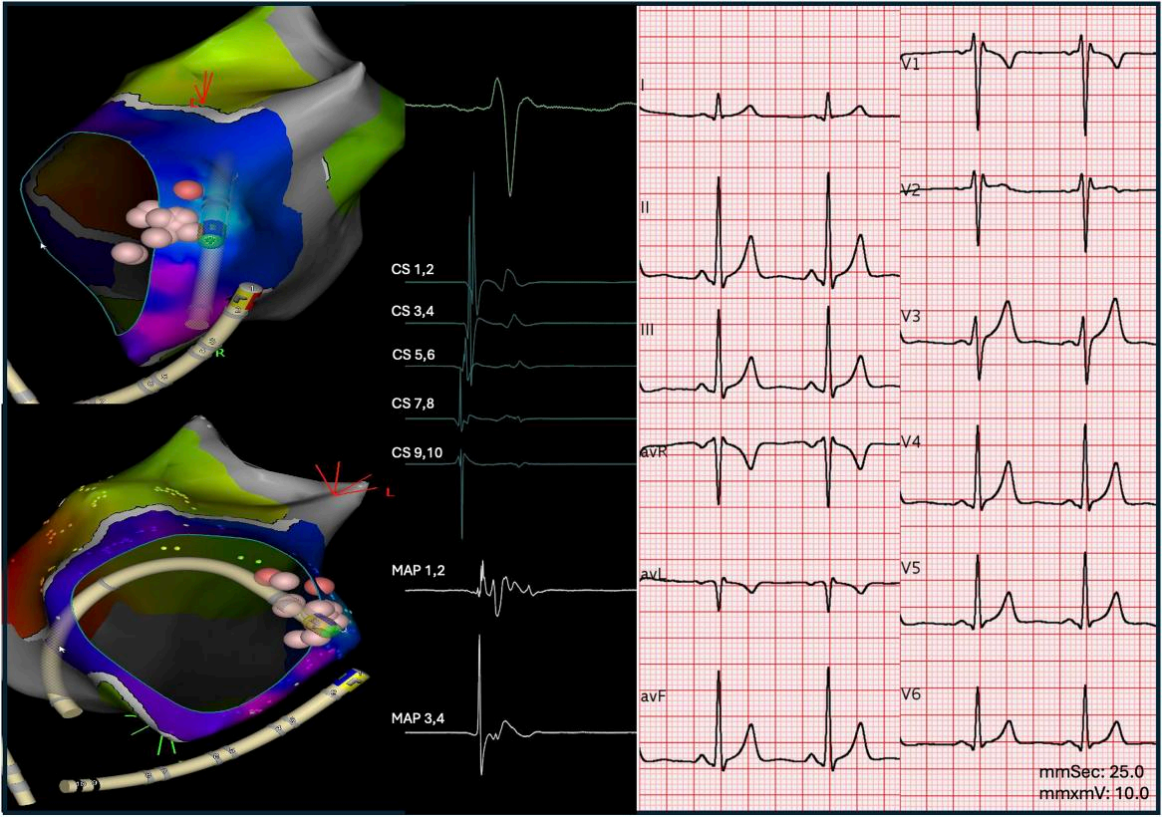
On the left panel: ablation tags are shown on the lateral aspect of the mitral annulus. The electrogram recorded near the ablation site is displayed on the distal dipole of the ablation catheter (MAP 1–2). On the right panel, the post-ablation ECG is comparable to the pre-ablation tracing. The persistence of the slurred upstroke in V3, along with the continued presence of normal septal Q waves in the lateral leads—both consistent with the previous ECG—supports the diagnosis of a latent accessory pathway. CS: coronary sinus; MAP: mapping catheter.

Due to the high-risk nature of the AP, a transseptal puncture was performed, followed by left atrial mapping with the CARTO system (Biosense Webster, Irvine, CA, USA). Ablation using a Thermocool SmartTouch SF (Biosense Webster, Irvine, CA, USA) irrigated catheter was performed during atrial pacing, achieving block after 15 s of radiofrequency; additional lesions were delivered (*Figure 5*). The final QRS complex showed no significant changes, with a persistent V3 slurred upstroke, but the AP was no longer evident, with neither antegrade nor retrograde conduction during pacing manoeuvres, not even after adenosine administration (Final ECG in *Figure 5*). No further arrhythmias were induced during programmed atrial stimulation. Genetic testing revealed no pathogenic mutations. Given the identification of a reversible cause of cardiac arrest, ICD implantation was avoided.

The patient was discharged, and at the 8-month follow-up, no arrhythmic recurrences were observed. The patient re-engaged in light physical activity with no associated symptoms.

## Discussion

This case illustrates an example of aborted cardiac arrest in a patient with no signs of structural heart disease. Previous routine medical examinations reported normal ECGs.

Performing an ECG after ROSC is a Class I recommendation according to the latest European guidelines.^1^ However, the specificity of ECGs in the first minutes after ROSC is limited, as ST-segment alterations are common in this phase, and transient ST-segment elevations may carry a high false-positive risk.^2^ In this case, ischaemia was ruled out with a coronary angiography, which showed normal coronary arteries.^3^

Idiopathic VF in the absence of overt structural or electrical abnormalities requires a stepwise diagnostic workflow to exclude concealed causes before labelling the event as truly idiopathic.^4^ High-resolution imaging, pharmacologic provocation tests for channelopathies (e.g. Brugada syndrome, long QT), and genetic screening are part of this evaluation.

Along with other causes of idiopathic VF, the presence of an AP should be considered, as even latent or subtle pre-excitation may still be malignant, possibly explaining the cause of SCA in our patient. These considerations led to the administration of adenosine, which confirmed antegrade conduction over a left lateral AP.^5,6^ Left-sided APs may present intermittently, latently, or with only subtle signs of pre-excitation, making their identification challenging on a 12-lead ECG. Their conductive properties can change with age,^7^ making the risk of SCA difficult to predict. In young individuals, AV nodal conduction may be fast enough to prevail and mask antegrade conduction over the AP, and unchanged QRS after the ablation supports this mechanism. Adenosine challenge can unmask this condition and prompt risk stratification through an invasive electrophysiological study,^6^ as in our case. Although considered to be low-risk, adenosine administration can trigger pre-excited AF, and appropriate caution is warranted.

In this case, a reversible cause of SCA was confirmed by the induction of haemodynamic collapse during pre-excited AF and was subsequently eliminated by catheter ablation, avoiding ICD implantation.

Current diagnostic algorithms for SCA survivors do not incorporate the adenosine administration test in every patient,^1,8^ but it should be noted that AV nodal conduction may mask a high-risk AP, and a normal QRS cannot exclude the presence of a latent one.^9^

ICD implantation is recommended in patients with documented VF in the absence of reversible causes.^1^ However, in young patients, ICD implantation carries a higher risk of long-term complications, including inappropriate shocks, psychological distress, lead fractures, and infections. In the present case, the identification of a reversible cause led to a shared decision not to proceed with ICD implantation. At the 8-month follow-up, the patient remained asymptomatic with no arrhythmic recurrences; however, long-term follow-up is necessary.

## Conclusions

In this case, the ST-segment elevation following ROSC proved to be misleading. In the diagnostic workflow, both first- and second-line tests yielded negative results, while adenosine administration successfully unmasked the presence of a latent AP, which would otherwise have remained undetected. Although not routinely performed in SCA survivors, this low-risk test can identify reversible causes of SCA and help to avoid unnecessary ICD implantations.

## Lead author biography


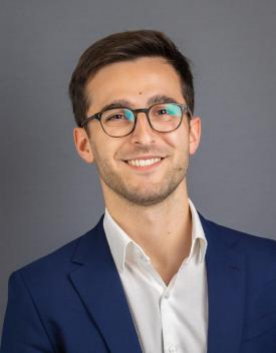


My name is Simone Taddeucci, and I am a young Italian electrophysiologist. I graduated from the University of Pisa in 2019 and completed my cardiology residency at the University of Siena. My professional activities are focused on interventional electrophysiology and cardiac pacing. I collaborated with the electrophysiology team at San Cataldo Hospital (Fondazione Toscana Gabriele Monasterio) in Pisa, where I developed my skills and contributed to several research projects. Following a brief fellowship at Leiden University Medical Center, I am currently working as an arrhythmologist at Livorno Hospital in Tuscany.

## Data Availability

No custom code or specific software was used in the analysis of the data presented in this article. The data underlying this article will be shared on reasonable request to the corresponding author.

## Notes

**Consent:** Written informed consent for publication was obtained from the patient.

**Funding:** The study received funding from Fondazione Toscana Gabriele Monasterio.
